# Identification of a Male Sterile Candidate Gene in *Lilium* x *formolongi* and Transfer of the Gene to Easter Lily (*L. longiflorum*) via Hybridization

**DOI:** 10.3389/fpls.2022.914671

**Published:** 2022-06-29

**Authors:** Takahiro Moriyama, Daniel John Shea, Naoto Yokoi, Seiro Imakiire, Takaaki Saito, Hikaru Ohshima, Hina Saito, Satoru Okamoto, Eigo Fukai, Keiichi Okazaki

**Affiliations:** ^1^Laboratory Plant Breeding, Graduate School of Science and Technology, Niigata University, Niigata, Japan; ^2^Akita Prefectural Agriculture, Forestry and Fisheries Research Center, Agriculture Experimental Station, Akita, Japan; ^3^Fruit Tree and Flower Division, Kagoshima Prefectural Institute for Agricultural Development, Kagoshima, Japan

**Keywords:** Easter lily, hybridization, male sterility, marker assisted selection, RNA-seq

## Abstract

Pollen-free varieties are advantageous in promoting cut-flower production. In this study, we identified a candidate mutation which is responsible for pollen sterility in a strain of *Lilium* × *formolongi*, which was originally identified as a naturally occurred male-sterile plant in a seedling population. The pollen sterility occurred due to the degradation of pollen mother cells (PMCs) before meiotic cell division. Genetic analysis suggested that the male-sterile phenotype is attributed to one recessive locus. Transcriptome comparison between anthers of sterile and fertile plants in a segregated population identified a transcript that was expressed only in pollen-fertile plants, which is homologous to *TDF1* (*DEFECTIVE in TAPETAL DEVELOPMENT and FUNCTION1*) in Arabidopsis, a gene encoding a transcription factor AtMYB35 that is known as a key regulator of pollen development. Since *tdf1* mutant shows male sterility, we assumed that the absence transcript of the *TDF1*-like gene, named as *LflTDF1*, is the reason for pollen sterility observed in the mutant. A 30 kbp-long nanopore sequence read containing *LflTDF1* was obtained from a pollen-fertile accession. PCR analyses using primers designed from the sequence suggested that at least a 30kbp-long region containing *LflTDF1* was deleted or replaced by unknown sequence in the pollen-sterile mutant. Since the cross between *L*. × *formolongi* and Easter lily (*L. longiflorum*) is compatible, we successfully introgressed the male-sterile allele, designated as *lfltdf1*, to Easter lily. To our knowledge, this is the first report of molecular identification of a pollen-sterile candidate gene in lily. The identification and marker development of *LflTDF1* gene will assist pollen-free lily breeding of Easter lilies and other lilies.

## Introduction

The genus *Lilium* of the Liliaceae family includes more than 100 species among which several species are important ornamental plants (Nishikawa et al., 1999, 2001; Marasek-Ciolakowska et al., 2018). *L. longiflorum* is distributed in the Amami Islands, the Ryukyu Archipelago in Japan, and in the main island in Taiwan (Hiramatsu et al., 2001; Sakazono et al., 2009). The intraspecies crossbreeding program of this species has developed many cultivars with trumpet-shaped pure white flowers, and consequently, those cultivars became an indispensable flower for ceremonial occasions, so called Trumpet Lily or Easter Lily. *L*. × *formolongi* has been developed by the interspecific hybridization between the *L. formosanum* and *L. longiflorum* followed by continuous backcrossing with *L. longiflorum*. As a result, the progeny successfully combined broad leaves like *L. longiflorum* and the characteristics of *L. formosanum* blooming within a year after sowing the seeds (Shimizu, 1987). Commercial cultivars of *L*. × *formolongi* and *L. longiflorum* are propagated by seeds or bulbs, respectively. For the year-round cut-flower production, *L*. × *formolongi* is employed in July to October and *L. longiflorum* in November to June in Japan.

Male sterility (pollen-free) benefits the ornamental crops (Smith et al., 2004; García-Sogo et al., 2010; Roque et al., 2019). Pollen is problematic in the case of flower arrangement since it stains perianths, clothes, etc. Therefore, florists have to manually remove the anthers immediately after flowering. Male-sterile flowers, on the contrary, do not cause seed formation by self-pollination, saving energy and resources for fruit production. This will extend the life of the flower. In addition, pollen sterility helps eliminate pollen allergens and avoid gene transfer from genetically modified crops into the ecosystem. Thus, pollen-free varieties are advantageous in promoting ornamental flower production. Recently, several pollen-free lilies are distributed into the commercial market, but as far as we know, pollen-free Easter lily cultivars are not available.

In plants, the pollen formation process is controlled by many genes, suggesting that the process can be easily impaired by a small number of mutations. Indeed, many mutants which have defects in microsporogenesis have been identified. Previous studies using those mutants in Arabidopsis revealed a gene network involving five transcription factors (TFs), DYSFUNCTIONAL TAPETUM1 (DYT1) (Zhang et al., 2006) and ABORTED MICROSPORES (AMS) (Sorensen et al., 2003), DEFECTIVE in TAPETAL DEVELOPMENT and FUNCTION1 (TDF1) (Zhu et al., 2008), MS188 (Zhang et al., 2007), and MALE STERILITY1 (MS1) (Ito et al., 2007). The interactive order in the regulatory network, DYT1–TDF1–AMS–MS188–MS1, is likely to be conserved in different species (Zhu et al., 2008; Cai et al., 2015; Gómez et al., 2015). Thus far, however, functional analysis of TDF1 has been limited to the three plant species including Arabidopsis, as follows. Cai et al. (2015) demonstrated that *OsTDF1* in rice compensates the pollen sterility when transformed into Arabidopsis *tdf1* mutant, suggesting that they are functionally orthologous. Recent studies reported that a deletion of the *TDF1* homolog (*AoMYB35*) in asparagus causes female flower due to defects in the anther development (Tsugama et al., 2017; Harkess et al., 2020).

The mutations of the TFs related to pollen development are classified into genic male sterility (GMS) and recessively inherited in most cases. Gametophytic GMS provides 50% male sterility by crossing a male-sterile line (rr, recessive homozygous male-sterile genotype) with a heterozygous individual (Rr). In many ornamental plants, since they can be clonally propagated, GMS is a useful source of male sterility. However, since the inheritance of male-sterile lilies has not been analyzed, it is difficult to effectively breed pollen-free varieties based on the inheritance manner. In this study, we report the identification of a male-sterile mutant and the responsible gene candidate in a pollen-free *L*. × *formolongi* plant AR01, later registered as a cultivar “Akita Kiyohime,” that naturally occurred in a seedling population.

## Materials and Methods

### Plant Materials

**Figure 1:**
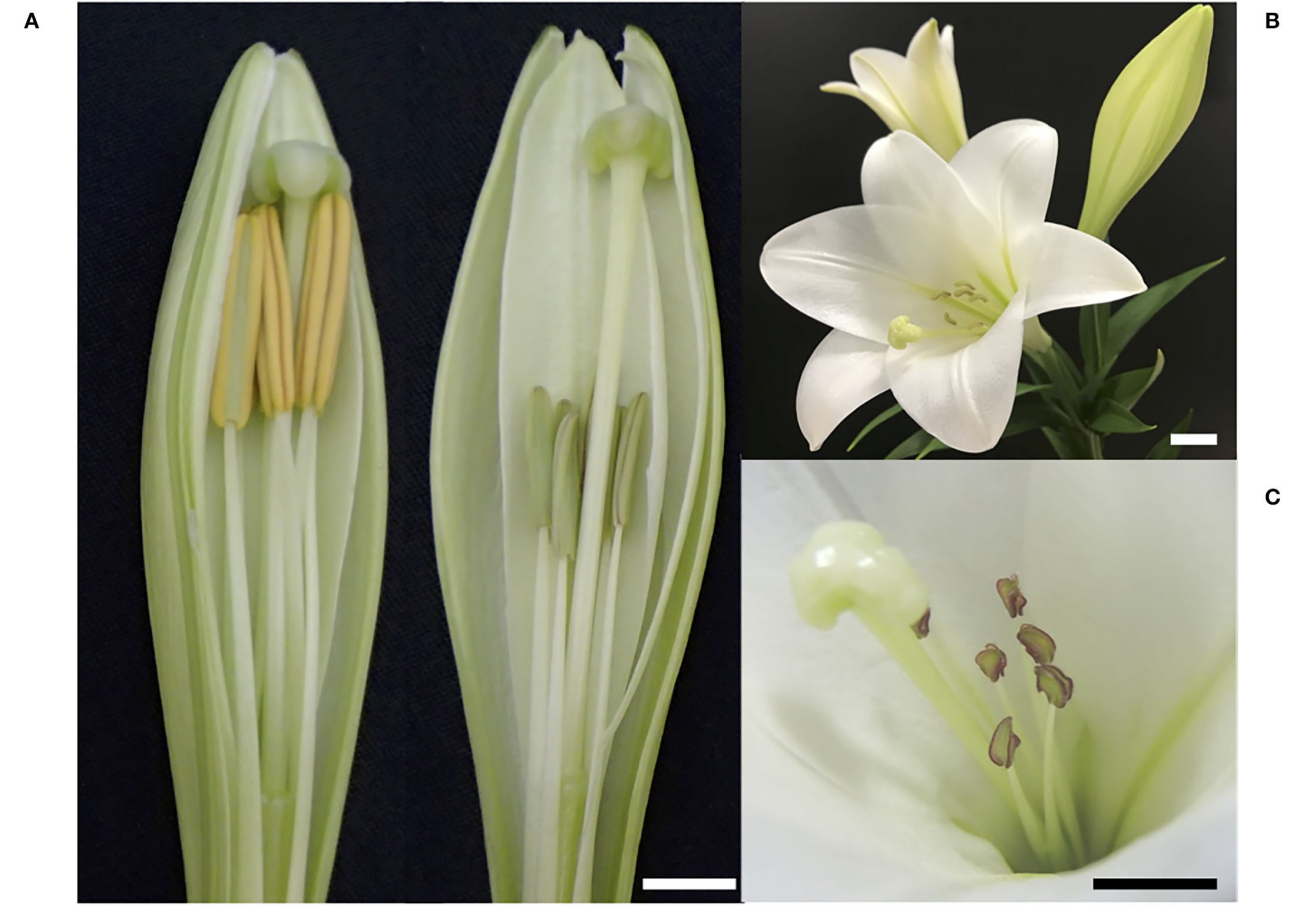
Appearance of pollen-fertile and sterile *L*. × *formolongi* plants. **(A)** Flower buds of a pollen-fertile plant (left) and the pollen-sterile AR01 plant (right). **(B)** An opened flower of AR01. **(C)** Close up of sterile anthers of AR01. Bar = 1 cm.

Several F_1_ plants were made from the cross between *L*. × *formolongi* cv. Hatsuki and cv. Raizan 2go (Supplementary Figure 1). *L*. × *formolongi* is self-compatible, but after the sib cross of F_1_ plants (22-1 and 23-1), a pollen-sterile plant was selected in the next generation and named PL01. PL01 was propagated by bulbs and repeatedly used as the founder of the subsequent pollen-sterile lines. PL01 was crossed with the selected pollen-fertile sib lines (18-3, 42-3, and 97-1) to obtain the segregating populations of pollen-fertile and sterile segregants. Each individual in the segregating population was cloned by bulblet propagation and thereafter used for RNA seq, genetic analysis, and anatomical observation. In the subsequent progeny, a pollen-sterile plant, AR01, was selected (Figure 1) and later registered as a cultivar “Akita Kiyohime.” F_1_ plants were made from the cross of AR01 and *L. longiflorum* cultivars (Cristal Horn, White Fox, and Pure Horn) and sibling cross-population made between F_1_ plants were used for DNA marker validation.

Pollen-sterile lines #72 and #318 developed by gamma-irradiated breeding were also used for paraffin section observation to compare pollen degradation processes between different pollen-sterile genotypes. In addition, sequence analysis of *Lilium TDF1* orthologs was conducted using a single plant of the following accessions, a pollen-fertile segregant of *L*. × *formolongi, L. longiflorum* cv. Hinomoto, Oriental hybrid lily cv. Siberia (*Lilium* spp.), and *L. formosanum* collected in a habitat of Niigata prefecture.

### Genetic Analysis

PL1607 population was made by crossing PL01 as female with pollen-fertile line 97-1 (inferred to be heterozygous at the locus responsible for the pollen phenotype) (Supplementary Figure 1). PL1620 population was made by the cross between the pollen-fertile lines, 83-2 × 220-1, where both lines were inferred to be heterozygous at the locus responsible for the pollen phenotype. The obtained seeds were planted in a cell tray containing regular soil, subjected to low-temperature treatment at 4°C for 2 weeks, and then raised in a greenhouse. After the seedlings grew at the 3–4 leaf stages, they were transplanted to a field at the end of April and the phenotypic separation of the presence or absence of pollen at the time of flowering was examined.

### Anatomical Observation of Anthers

Flower buds (13 to 26 mm in length) were collected from pollen-sterile and pollen-fertile segregants in the segregating population, 26–47. Microsporogenesis of sterile lines, #72 and #318, developed by the gamma irradiation breeding, was also collected. The collected buds containing all six anthers were immersed in Carnoy's solution (ethanol: acetic acid = 3: 1) at room temperature for 12 h. Then, the samples were dehydrated using alcohol series. They were stored in chloroform and embedded in paraffin. Transverse sections were made at a thickness of ~12 μm and deparaffinized with Histo-clear and ethanol of gradient concentration. The tissues were stained with toluidine blue (0.05 %) and dehydrated in ethanol series (50–100 %) and Histo-clear. Finally, the sections were mounted in Canada balsam and observed by light microscopy (BX-60; SZX7, Olympus, Tokyo, Japan) and photographed using a CCD camera (DP60, Olympus, Tokyo, Japan).

### Transcriptome and GO-Enrichment Analysis

For RNA sequencing, anthers were collected from pollen-sterile and pollen-fertile segregants of the segregating population, 26–47, where the flower buds of lengths 11, 12, and 14 mm were collected from three different plants of pollen-sterile and pollen-fertile segregants, respectively. The anthers were then excised from the three different-sized buds and mixed separately with pollen-sterile and pollen-fertile bulk sampling. The anthers were instantly frozen in liquid nitrogen and stored in the deep freezer till use. The samples were crushed with a pestle, and RNA was extracted using RNeasy Plant Mini Kit (QIAGEN) according to the protocol. RNA samples were sequenced by GeneBay, Inc., Japan, using Illumina HiSeq 2000 platform and dUTP-based directional sequencing method. The obtained 150 bp-long paired-end reads from the two cDNA libraries were mixed and *de novo* assembled with TRINITY, to obtain reference transcript sequences (TCs). A *de novo* assembly was performed using the Trinity pipeline and transcript quantification was conducted as described by Haas et al. (2013). Then, the reads were separately mapped in the two cDNA libraries of the pollen-sterile and pollen-fertile plants. Fragments Per Kilobase of transcript per Million (FPKM) mapped reads were used to indicate the expression abundance of respective unigene, and those with a transcription amount of |logFC|>10 at the level of FDR <0.05 were defined as differentially expressed transcripts (DETs). The most homologous protein in Arabidopsis to each DET was determined by BLASTX using BLAST+ (ncbi-blast-2.9.0+) and protein sequences in Arabidopsis as database (Araport11_genes.201606.pep.fasta, https://www.arabidopsis.org/download/index-auto.jsp?dir=%2Fdownload_files%2FSequences%2FAraport11_blastsets%2FArchived%2FJune_2021). GO-enrichment analysis was performed using the list of homologous Arabidopsis gene names as a query on https://www.arabidopsis.org/tools/go_term_enrichment.jsp.

### RT-PCR and qRT-PCR

Flower buds of lengths 10–30 mm of pollen-fertile and sterile segregants were collected from the segregating population, 26–47. Then, anthers, stigmas, and perianths were separated from each bud, instantly frozen in liquid nitrogen, and stored at −80°C until use. Leaves were collected from adult plants. The total RNA was extracted using RNeasy Plant Mini Kit and RNA-Free DNase Kit (QIAGEN) according to the manufacturer's protocol. Then, cDNA was synthesized using PrimeScript 1st strand cDNA Synthesis Kit (Takara Bio, Shiga, Japan). The RT-PCR products were electrophoresed on a 1% agarose gel, stained with ethidium bromide.

qRT-PCR mixture was prepared according to the protocol of iTaq ™ Universal SYBR Green One-Step Kit (Bio-Rad). For the relative quantification of the *LflTDF1* transcripts, qRT-PCR reaction was performed with LightCycler Nano (Roche Diagnostics, Basel, Switzerland). According to the published protocol, the first step was performed at 50°C for 10 min, the second step was performed at 90°C for 1 min, and the three-step amplification reaction was performed at 95°C for 15 s and 60°C for 60 s for 45 cycles. In the melting reaction, the temperature was raised from 60°C to 97°C at a pace of 0.1°C per second, and the PCR results were analyzed using LightCycler Nano Software.

Primers used to detect *LflTDF1* transcripts were LflTDF1- f2 and LflTDF1- r2. *Lilium* actin gene was used as the internal control. Primer pair used to detect *Lilium* actin transcripts was LhACTIN (Sakai et al., 2019). Sequences of the primers are listed in Supplementary Table 1.

### Genomic DNA Extraction and Marker Assay

Leaf tissue of about 1 cm in diameter was collected, placed in a 1.5-ml plastic tube, and frozen with liquid nitrogen for DNA extraction. DNA extraction for genotyping was followed by the CTAB method in accordance with Sato et al. (2019). For DNA sequencing, plant DNA was isolated using NucleoSpin Plant II Kit (Takara Bio, Shiga, Japan).

DNA fragments of *TDF1* allele and the flanking region were amplified with 100 ng of template DNA in a 10 μl reaction volume containing 2 pmol of each primer and 1× EmeraldAmp PCR Master Mix (Takara Bio, Shiga, Japan). Thermal cycling conditions included denaturation at 95°C for 2 min, followed by 35 cycles of denaturation at 95°C for 30 s, annealing at 53–60°C (depending on the melting temperature of primers) for 30 s, and extension at 72°C for 1 min, and a final extension at 72°C for 5 min. The allele-specific primers and the sequence primers are listed in Supplementary Table 1. Unigene (15801) primer pair was used for a positive control (Lang et al., 2015). PCR products were separated by electrophoresis on 1.0% agarose gel and visualized under a UV imaging system. The F_1_ plants were made by crossing AR01 with three *L*. *longiflorum* cultivars, Cristal Horn, White Fox, and Pure Horn, and then, sibling crosses among F_1_ plants were conducted to produce the subsequent progenies. A total of 31 pollen-sterile plants and seven pollen-fertile plants in those progenies were used for genotyping. For the genotyping of *L*. × *formolongi*, the segregating population of PL1607 and PL1620 was used. In addition, 24 pollen-sterile plants and seven pollen-fertile plants were selected from the segregating population, 26–47.

### DNA Sequencing

For sequence analysis of the *TDF1* homolog gene of *Lilium* species, after fractionating the PCR products by 1% agarose gel electrophoresis, the portion of the target band was cut out and purified using FastGene Gel/PCR Extraction Kit (NIPPON Genetics, Tokyo, Japan). The purified PCR products were cloned into T-Vector pMD20 (Takara Bio Inc., Shiga, Japan). Three clones per each sample were sequenced by Fasmac Co. Ltd. (Kanagawa, Japan). The obtained sequences were analyzed using sequence analysis software, GENETYX v.12 (Genetyx Corporation, Tokyo, Japan) and Sequencher v 2.0 (Hitachi Software, Tokyo, Japan). The genomic sequences of the alleles collected in this study were aligned using MEGA 7 (Kumar et al., 2016). The phylogenetic relationship was inferred by using the maximum likelihood method (Hasegawa et al., 1985) added to MEGA 7.

### Nanopore Sequencing and Genomic PCR in the *LflTDF1* Region

Nanopore sequencing was conducted in a pollen-fertile plant, N-1, selected from a seedling population of *L*. × *formolongi*. DNA was isolated by NucleoBond HMW DNA isolation kit (TaKaRa Bio Inc., Shiga, Japan). The DNA sequencing was conducted by GeneBay Co., Ltd., (Kanagawa, Japan) using PromethION. The nanopore read sequence harboring *LflTDF1* was corrected by the short read of *L*. × *formolongi* cv. Augusta obtained by Hiseq 2000 device. The PCR experiment using six primer pairs designed based on the nanopore sequence (contig #1) encompassing the *LflTDF1* region was conducted in five pollen-fertile genotypes including N-1, the pollen-fertile segregant (63-1 and 97-2), the originator's line of AR01 (Raizan 2go) and cv. Augusta, as well as in the pollen-sterile AR01.

## Results

### Genetic Analysis

A sibling cross of F_1_ plants made from the cross between *L*. × *formolongi* cv. Hatsuki and cv. Raizan 2go produced the pollen-sterile line, PL01. Then, we made the segregating populations, PL1620 and PL1607 using PL01 and its progeny, and investigated the inheritance manner of the pollen-sterile gene (Supplementary Figure 1). The PL1620 population was made by the cross between the pollen-fertile lines, 83-2 × 220-1, where both lines were inferred to be heterozygous at the locus responsible for the pollen phenotype. The phenotype assay of PL1620 revealed that the phenotyping in the progeny fit to 3 (fertile):1 (sterile) ratio by chi-square test (Table 1), indicating that both parents are heterozygous (*LflTDF1*/*lfltdf1*).

**Table 1:** Chi-square test for segregation of pollen-fertile and sterile plants in the segregating populations.

**Population**	**Crossing (genotype tested)**	**No. of plants**	**No. of pollen fertile plants**	**No. of pollen sterile plants**	**Chi-square vale (probability)**
PL1620	Fertile × Fertile (*LflTDF1*/*lfltdf1* × *LflTDF1*/*lfltdf1*)	37	30	7	3.2 (0.3<p<0.5)
PL1607	Sterile × Fertile (*lfltdf1*/*lfltdf1* × *LflTDF1*/*lfltdf1*)	46	28	18	2.1 (0.1<p<0.2)

The PL1607 population was made from crossing the pollen-sterile PL01 line with the pollen fertile 97-1 inferred to be heterozygous at the locus responsible for the pollen phenotype. The seed set rate of PL01 plant was high (305 seeds/pod), indicating that the female fertility of the pollen-sterile mutant is normal. In this population, the phenotyping data matched the 1: 1 ratio by chi-square test, which fit to a single recessive gene model (Table 1). Thus, since the genetic analysis in the two segregating populations suggested that the genetic control of the male-sterile phenotype is under a single recessive gene, we applied RNA-sequencing technology to identify a presence/absence gene expression between pollen-fertile and sterile plants.

### Anatomy of Microsporogenesis

**Figure 2:**
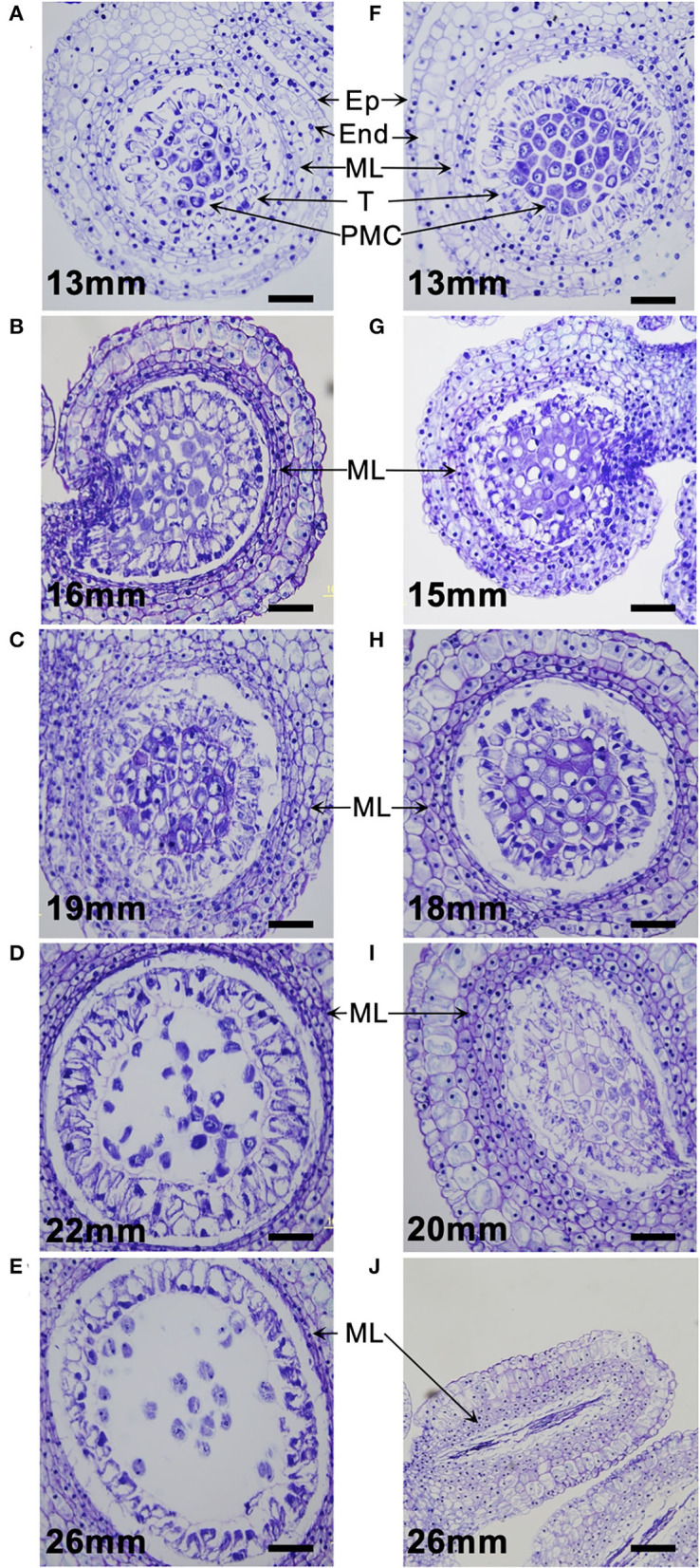
Microsporogenesis of *L*. × *formolongi*. **(A–E)** Pollen-fertile segregants. **(F–J)** Pollen-sterile segregants. Ep, epidermis; End, endothecium; ML, middle layers; T, tapetum; PMC, pollen mother cells. Bar = 100 μm.

Paraffin section observation revealed that in pollen-fertile segregants of the segregating population (26–47), pollen developmental stages were associated with the size of flower buds: 10–18 mm in the PMC proliferation stage, 20–22 mm in the premeiotic stage, 23–28 mm in the meiotic cell division stage, and >29 mm in the uninuclear and binuclear microspore stage (Figures 2A–E). In the pollen-sterile segregants, PMCs were normally proliferated but could not start meiotic division in PMCs in the flower bud length 20 mm, and then those PMCs were degraded in the premeiotic stage (Figures 2F–J). At this stage, the middle layers of the anther of the pollen-sterile segregants were densely stained and became swollen (Figures 2H,I). Paraffin section observation of the *lfltdf1* mutant confirmed no production of pollen debris in the anther. This can be explainable for the appearance of perfect pollen-free anthers in the blooming flowers of *lfltdf1* mutant (Figure 1).

The pollen-sterile lines, #72 and #318, developed by the gamma-irradiated breeding normally underwent the microsporogenesis up to the premeiotic stage where the PMCs became free due to callose degeneration in the locule (Supplementary Figures 2A,C) like the pollen-fertile plant (Figure 2D), but failed meiotic cell division (Supplementary Figures 2B,D). The aberration in the middle stage of meiosis produced many debris in the anther locule. This pollen debris remained in some anthers attached to the blooming flowers (Supplementary Figures 2E,F).

### RNA-Seq and GO Analysis

Illumina sequencing of RNA extracted from anthers of the pollen-sterile and pollen-fertile segregants in the segregating population, 26–47, and the following Trinity assembly obtained 56,576 transcripts (isoforms). Of them, 253 were identified as differentially expressed transcripts (DETs) between pollen fertile (p) and sterile (np) by statistical analysis using edgeR of which FDR was <0.05, with 97 DETs in p>np (<-10 logFC) and 156 DETs in p<np (>10 logFC), respectively (Table 2 and Supplementary Table 2). A blastx search using the 253 TCs as query and Arabidopsis protein sequences as a database revealed that 11 DETs (five in p>np and six in p<np) did not match any protein sequences in the Arabidopsis. On the contrary, there were 242 DETs with hits (92 in p>np and 150 in p<np). The list of identified Arabidopsis homologs was subjected to GO-enrichment analysis using the Arabidopsis platform available at TAIR website (https://www.arabidopsis.org/tools/go_term_enrichment.jsp). The “gene expression” was detected as an over-represented GO term in the list of Arabidopsis homologs of p<np DETs. On the contrary, six over-represented GO terms, which were all related to sexual reproduction in the biological process, were found in the list of Arabidopsis homologs of p>np DETs (Table 3 and Supplementary Table 3).

**Table 2:** Differential expression analysis in the anthers (collected from the bud size, 11–14 mm) of pollen-sterile (np) and fertile (p) plants with the criteria of FDR < 0.05 and | logFC|>10, best-hit blastx homology search, and GO analysis.

		**Blastx**		
**DETs category**	**No. of DETs**	**Homolog**	**No homolog**	**Significantly enriched GO term**
p>np	97	92	5	7
p<np	156	150	6	1
total	253	242	11	8

**Table 3:** Significantly enriched GO terms in differentially expressed transcripts (DETs) identified in the anthers (collected from the bud size, 11–14 mm) of pollen-sterile (np) and fertile (p) plants.

**DETs category**	**GO Type**	**GO Term**	**Gene number**	**Relative expression**	**P-value**
p>np	BP	Floral organ development	8	10.17	4.46E-03
		Reproductive structure development	15	3.87	2.06E-02
		Reproductive system development	15	3.86	2.10E-02
		Developmental process involved in reproduction	16	3.44	4.12E-02
		Reproductive process	18	3.22	2.70E-02
		Reproduction	18	3.2	2.91E-02
	MF	Protein binding	36	2.06	1.01E-02
*p* < np	BP	Gene expression	15	4.17	5.62E-03

One of the DETs included in the over-represented GO terms, which was expressed only in the pollen-fertile plants but not in the pollen-sterile plants, was identified as a homolog of *Arabidopsis thaliana TDF1* (AtMYB35) which belongs to the member of the R2R3 factor gene family. Since Arabidopsis *TDF1* mutant shows male sterility, we further analyzed the identified DET as a candidate in *L*. × *formolongi*, designated as *LflTDF1*.

### RT-PCR Analysis

**Figure 3:**
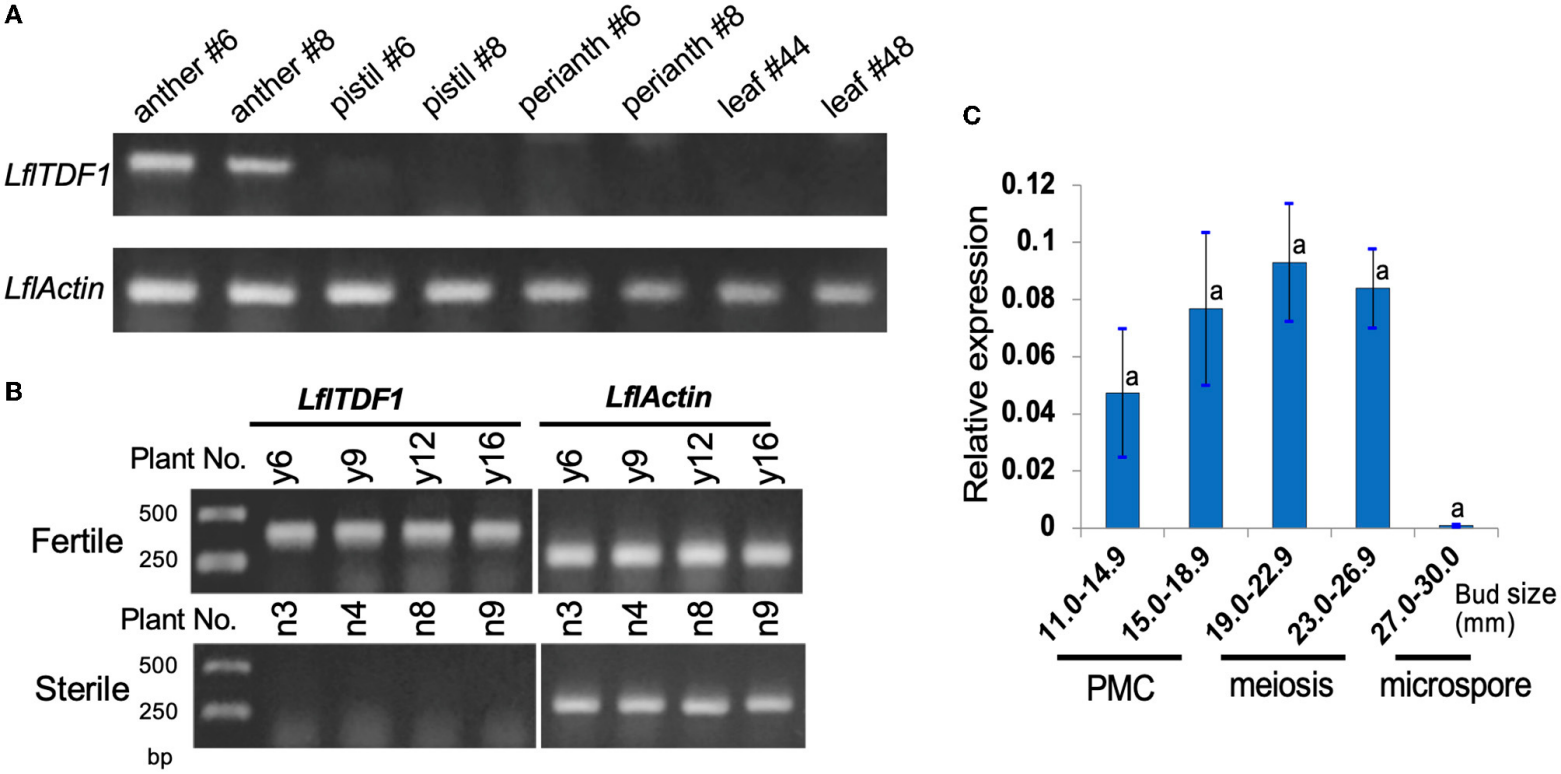
Expression analysis of *LflTDF1* gene by RT-PCR and qRT-PCR. The samples were collected from the population derived from the cross of pollen-sterile and pollen-fertile segregants. Actin gene of *L*. × *formolongi* was used as the internal control. **(A)** Tissue-specific expression of *LflTDF1* in the pollen-fertile segregants. The samples were collected from the two different plants indicated by #. **(B)** The expression of *LflTDF1* in the anthers (collected from the bud size 14–26 mm) of the pollen-fertile and sterile segregants. Four different samples of the pollen-sterile and pollen-fertile segregants were shown. **(C)** qRT-PCR detected the expression of *LflTDF1* in the anthers collected from the buds of pollen-fertile segregants having different sizes. The pollen developmental stages are referred in Figure 2. Error bar represents standard deviation, and means were tested by Tukey's test at P = 0.05.

RT-PCR was performed using anthers, stigmas, perianths, and leaves of two pollen-fertile segregants in the segregating population (26–47) (Figure 3A). As a result, RT-PCR revealed that *LflTDF1* mRNA was expressed only in anthers but not in the pistils, perianths, and leaves. The anther-specific *LflTDF1* mRNA was expressed only in pollen-fertile segregants but not in pollen-sterile segregants (Figure 3B), which is consistent with the RNA-seq data. The RT-PCR of *LflTDF1* mRNA using the developing anthers with different pollen developmental stages revealed that the transcription levels were too low to detect at 30 mm bud length, while the expression started from a flower bud length of 11 mm and tended to reach a peak at the meiotic division stage of flower bud length of 19.0–22.9 mm (Figure 3C). Therefore, we concluded that the *LflTDF1* allele of the pollen-sterile line has lost its transcription activity in the anthers. Thus, we designated this pollen sterility allele as *lfltdf1*.

### DNA Sequence Analysis of *LflTDF1*

**Figure 4:**
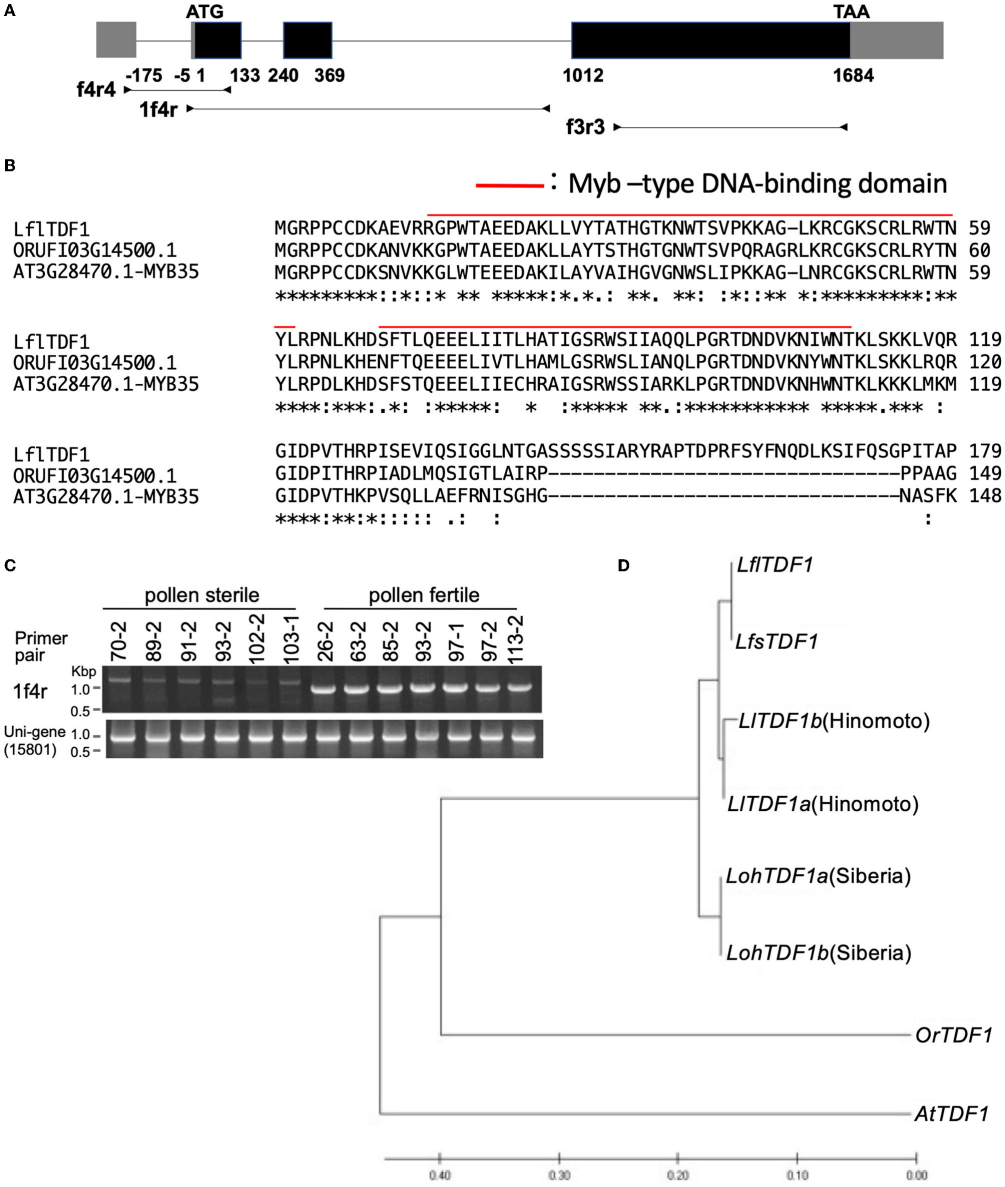
**(A)** Schematic diagram of the *LflTDF1* gene structure including exons (boxes) and introns (horizontal line). Gray and black boxes indicate untranslated and CDS region, respectively. Horizontal lines with attached arrowheads indicate PCR amplicon regions for genotyping with the intragenic markers. **(B)** Comparison of partial amino acid sequences of *TDF1* orthologs in *L*. × *formolongi, Oryza rufipogon* (ORUFI03G14500.1), and *Arabidopsis thaliana* (AT3G28470.1), respectively. Red line indicates Myb–type DNA-binding domain predicted by Pfam. **(C)** An example of genotyping of *LflTDF1* intragenic dominant marker, 1f4r, using the segregating population (26–47). Unigene (15801) primer pair was used for a positive control. **(D)** Phylogenic relationships of *TDF1* orthologs in *L*. × *formolongi* (*LflTDF1*), *L. formosanum* (*LfsTDF1*), *L. longiflorum* cv. Hinomoto (*LlTDF1*), *L*. spp. Oriental hybrid cv. Siberia (*LohTDF1*), *Oryza rufipogon* (*OrTDF1*, ORUFI03G14500.1), and *A. thaliana* (*AtTDF1*, AT3G28470.1). The phylogenetic tree was inferred by using the maximum likelihood method added to MEGA 7.

*LflTDF1* transcript and the corresponding genomic locus were sequenced in a pollen-fertile plant in the segregating population (26–47). Comparison of the transcript sequences and genomic sequences confirmed the location of four exons and three introns in the *LflTDF1* gene where an exon locates in the 5'-UTR and three exons in the CDS (Figure 4A). *TDF1* orthologs were amplified from a single plant of *L*. × *formolongi, L. longiflorum* cv. Hinomoto, *L. formosanum*, and Oriental hybrid lily cv. Siberia to determine the nucleotide sequences. As a result, the alignment of 2287bp including 5'- and 3'-UTR identified a single allele (homozygous) in each *L*. × *formolongi* and *L. formosanum*, and two alleles (heterozygous) in each cv. Hinomoto and cv. Siberia (Supplementary Table 4). Therefore, the two alleles of *TDF1* of Easter lily and Oriental hybrid lily were designated as *TDF1a* and *TDF1b* (Figure 4D). *LflTDF1* is composed of 311 amino acids (aa), and the N-terminal region containing the myb-type helix-turn-helix (HTH) domain was conserved among the distantly related species such as *Oryza rufipogon* and *A. thaliana* (Figure 4B), suggesting that the *LflTDF1* acts as a MYB transcription factor. The aa sequences after the conserved region showed low similarity among the different species (Supplementary Figure 3). Since the pollen fertility of the wild and mutant type is determined by the presence/absence of *LflTDF1* gene, we designed some intragenic markers to detect *LflTDF1* gene in the segregating population (Figure 4A). *LflTDF1* intragenic dominant marker, 1f4r, clearly detected the presence/absence of *LflTDF1* gene in the segregating population (26–47) (Figure 4C). Phylogenic analysis revealed that *L. longiflorum* and *L. formosanum* are monophyletic and Oriental hybrid lily cv. Siberia was included in the other clade (Figure 4D). The *LflTDF1* showed higher identity with the orthologous *LfsTDF1* gene of *L. formosanum*, a parental species of *L*. × *formolongi*, than that of *L. longiflorum* (Easter lily), the other parental species. The result suggests that *LflTDF1* originated from *L. formosanum* in the interspecific breeding process.

### Correlation of Phenotypes and Genotypes

As described above, the genetic analysis in the segregating populations of PL1620 and PL1607 suggested that the genetic control of the male-sterile phenotype is under a single recessive gene. The genotype of cross combination of PL1620 and PL1607 suggested to be *LflTDF1*/*lfltdf1* × *LflTDF1*/*lfltdf1* and *lfltdf1*/*lfltdf1* × *LflTDF1*/*lfltdf1*, respectively (Table 4). An example of genotyping of LflTDF1 intragenic dominant marker, f4r4, using the segregating population is shown in Supplementary Figure 4. In the PL1620 population, the genotyping using the dominant marker LflTDF1-f4r4 was perfectly matched with the phenotyping data of the progeny. Genotyping in the PL1607 population was almost perfectly matched with the phenotyping data of the progeny, but there was one exceptional plant, which was pollen-sterile but produced the *LflTDF1* specific marker band in the PCR test. We could not re-examine this exceptional plant in the next growing season due to the rotting of the bulb.

**Table 4:** Phenotyping and genotyping in the segregating populations and the selected breeding lines.

	**Population (genotype tested)**	**No. of plants**	**Phenotyping**	**Genotyping with the dominant marker LflTDF1-f4r4**		
			**No. of fertile plants**	**No. of sterile plants**	**Presence of the TDF1 marker band**	**Absence of the TDF1 marker band**
Segregating population	PL1620 (*LflTDF1*/*lfltdf1* × *LfTDF1*/*lftdf1*)	37	30	7	30	7
Segregating population	PL1607 (*lfltdf1*/*lfltdf1* × *LflTDF1*/*lfltdf1*)	46	28	18	29	17
Selected breeding lines	Progeny of PL01 × *L*. *longiflorum*	38	7	31	7	31
Selected breeding lines	Progeny of PL01 × *L*. × *formolongi*	31	7	24	7	24

Since the cross between *L*. *longiflorum* and *L*. × *formolongi* is compatible, the F_1_ plants were made by crossing AR01 with three *L*. *longiflorum* cultivars, Cristal Horn, White Fox, and Pure Horn. Then, sibling crosses among F_1_ plants produced the subsequent progenies, of which 31 pollen-sterile plants and seven pollen-fertile plants were used for genotyping. In addition, 24 pollen-sterile plants and seven pollen-fertile plants were selected from the segregating population (26–47) of *L*. × *formolongi*. In the selected plants, the dominant marker LflTDF1-f4r4 was perfectly matched with the phenotyping data of the progeny (Table 4).

### Nanopore Sequencing of *LflTDF1* Locus

**Figure 5:**
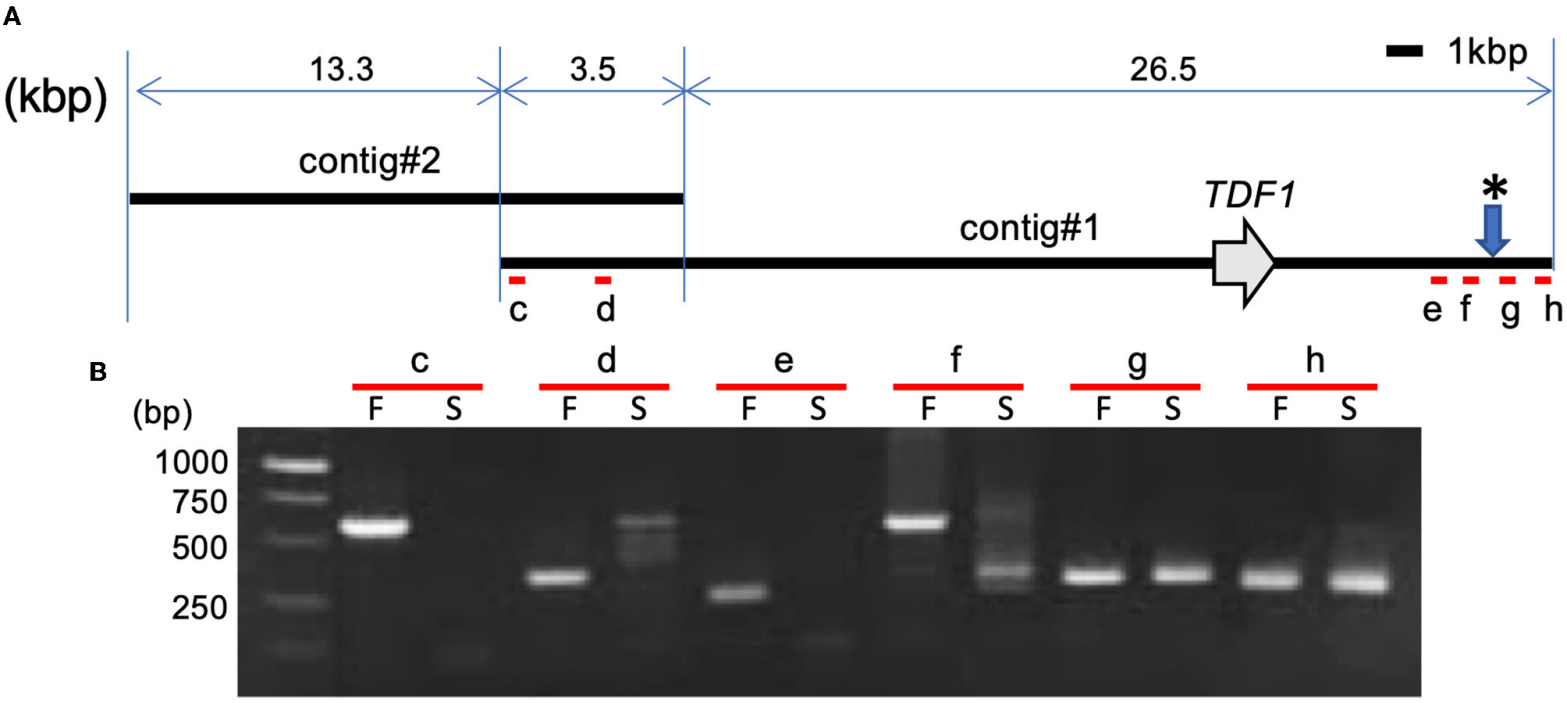
Structural analysis of *LflTDF1* flanking region. **(A)** Location of *LflTDF1* and nanopore sequencing contigs obtained from a pollen-fertile line. **(B)** PCR amplification in the pollen-fertile genotype (F) and sterile AR01 (S) using the primer pairs (c to h) that were designed within the contig #1. Since five pollen-fertile genotypes amplified the same sized amplicon in the respective primer set, the amplicon of one genotype is represented as an example. * indicates the chromosome breaking points inferred from the PCR amplification of each primer set.

The nanopore sequencing technology was applied to collect the *LflTDF1* harboring genomic region sequences in a pollen-fertile plant of a seedling population of *L*. × *formolongi*. The read yield was 68.74GB (>1 kbp). The number of reads and the average length were 4,520,860 (> 1 kbp) and 15.21 kbp, respectively. The *LflTDF1* region was only detected in the sequence (designated as contig #1), and no other sequences containing *LflTDF1* were found. Contig #1 overlapped contig #2 at the left end and repetitive sequences at right end, respectively (Figure 5 and Supplementary Figure 5). The homology of the overlapping region of contig #1 and contig #2 was not high (Supplementary Figure 5), indicating that the location of contig #2 is tentative.

The PCR experiment using six primer pairs in the genomic region encompassing the contig #1 was conducted in five pollen-fertile genotypes including the pollen-fertile segregants and the pollen-sterile AR01 (Figure 5). As a result, the primer pairs, c to f, located from the left end to the right end of contig #1, amplified the same sized fragment in all pollen-fertile genotypes but not in the pollen-sterile AR01. On the contrary, the primer pairs g and h were able to amplify the fragments in both pollen-fertile and sterile genotypes. Since those primer pairs were designed in the repetitive sequence located in the right end of contig #1, we confirmed whether the primer pair g can specifically amplify the linked region of *LflTDF1* gene using the segregation population, PL3105. The primer pair g PCR amplicons of the pollen-sterile AR01 and pollen-fertile lines were sequenced, so that there was an SNP at the *Eco*RV recognition site between the two lines and the segregating population (PL3105), that is, AR01 (*lfltdf1*/*lfltdf1*) × 26–47 (*LflTDF1*/*lfltdf1*) was evaluated for genotyping experiment. Segregation profile of *TDF1* and *tdf1* genotypes in this population using the intragenic dominant markers (f4r4) and the codominant CAPS linkage marker g was perfectly matched (Supplementary Figure 6). This result indicates that the linkage marker g could specifically amplify the flanking region of *LfTDF1*gene. Therefore, this data suggested that the chromosomal breaking point inferred from the PCR amplification of each primer set was between the two regions amplified by the primer pairs, f and g, respectively. Overall, the corresponding region in the pollen-sterile plants is likely deleted at least 30 kbp corresponding to almost whole length of contig #1 or replaced by an unknown sequence.

## Discussion

The transcriptome comparison using the developing anthers (10–14 mm) identified one of the DETs expressed only in pollen-fertile plants. This DET was identified as a homolog of *Arabidopsis thaliana TDF1* (*AtMYB35*). Genetic analysis in the segregating population showed that the pollen sterility of PL01 was inherited in a single recessive manner, and the dominant marker LflTDF1-f4r4 was almost perfectly matched with the phenotyping data of the progeny. The one exceptional plant was positive for the dominant marker LflTDF1-f4r4 test but was pollen sterile. This inconsistency may be due to the pollen sterility caused by physiological disorder that occasionally occurs under unsuitable circumstances. Alternatively, it is due to the sampling miss. The nanopore sequence indicates that AR01 is lacking *LflTDF1* region spanning at least 30 kbp or is replaced by unknown sequence. In addition to this evidence, since Arabidopsis *TDF1* mutant shows male sterility and *TDF1* is a key regulator of tapetal development and microsporogenesis, we identified *LflTDF1* as a candidate gene for the pollen sterility of AR01. One of the parental materials, cv. Hatsuki and cv. Raizan 2go (both male fertile cultivars), is probably heterozygous in the *LflTDF1* allele. However, it has not yet been determined which parent provided the mutant allele to the progeny.

The pollen sterility of Arabidopsis *tdf1* mutant is caused by dysfunction of anther wall layers including endothecium, middle layer, and tapetum (Zhu et al., 2008). Zhu et al. (2008) reported that in the *attdf1* mutant, cell vacuolation in both the epidermis and the endothecium started in the early anther development stage appearing pollen mother cells, followed by vacuolation of the middle layer at the premeiotic stage, and thereafter, the tapetum was vacuolated, hypertrophic, and multilayered. Finally, the swollen tapetum cells crush microspore. The similar anatomical feature of anther development of *ostdf1* mutant was reported by Cai et al. (2015). In contrast to *attdf1* and *ostdf1* mutant, the *lftdf1* genotype did not show vacuolation of the epidermis, endothecium, and middle layer in the early stage of PMC proliferation and showed no enlargement of the tapetum cells. On the contrary, the middle layer of the *lftdf1* genotype became dense and swollen, compared to the wild type, and then, the thick middle layer remained visible till the degradation of PMCs. It is known that *Lilium* anther wall layers preserve many starch grains consumed in the microspore formation (Clément et al., 1994; Clément and Audran, 1995). Therefore, the degradation of PMCs and tapetum in the meiotic stage prevents the transfer of starch degradation products to the tapetum, causing the deposition of the excessive glucose or its derivatives in the anther wall layer. This may be one of the reasons why the dense thick middle layers appeared in AR01. In both *attdf1* and rice *ostdf1* mutants, the tetrads were formed by meiosis, but the resulting tetrads were abnormally unreleased and not free in the locules. In contrast, the *lfltdf1* mutant never enters meiosis and the PMCs are degraded prior to meiosis, indicating that the time of appearance of anther development defects in the *tdf1* mutants may vary from species to species.

In pollen-sterile mutants #72 and #318 produced by gamma irradiation, meiosis began but was incomplete, so some anthers attached some pollen debris during flowering. These male meiotic mutants released PMCs in the anther locule like wild type (Supplementary Figures 2A,C) but the meiotic cell division failed, and the resulting aberrant PMC products might be harder, probably due to the deposition of sporopollenin like substance. In contrast, the pollen sterility caused by *lfltdf1* mutation never produces pollen debris during flowering. This is owing to the earlier degradation of PMCs prior to meiotic division in *lfltdf1* plant, where the aberrant premeiotic product completely disappeared in the anther locules. In this regard, the *lfltdf1* mutant is a superior breeding material for lily pollen-free breeding.

GO analysis performed with the p>np set of DET on the Arabidopsis basis identified 18 Arabidopsis genes such as U-box domain-containing protein 4 (PUB4) ortholog in biological process ontology (Supplementary Table 3). PUB4 plays an important role for controlling tapetum abortion (Wang et al., 2013), suggesting that many genes including microsporogenesis-related genes were directly/indirectly affected by lack of *LflTDF1* gene. In addition, this aberrant regulatory process in the mutant began early in the development of PMCs in which sample RNA was collected. This is quite before the pollen abortion stage (meiotic stage) of the mutant. Further studies using appropriate logFC criteria instead of ± 10 log FC used in this study are needed to learn more about the gene expression profile in the mutant, which helps to better understand the pollen development process of lilies.

We found two alleles of *TDF1* homologs in a single plant of each species, *L*. *longiflorum* cv. Hinomoto and an Oriental hybrid cv. Siberia. *L*. *longiflorum* and an Oriental hybrid lily cv. Siberia are heterozygous because of outcrossing due to self-incompatible nature, and therefore, the two polymorphic *TDF1* homologs come from outcrossing. On the contrary, a single *TDF1* sequence was identified in a single plant of the respective *L. formosanum* and *L*. × *formolongi*. This may be due to the self-compatible nature of the two species, which produce more homozygosity of *LflTDF1* locus. *LflTDF1* of *L*. × *formolongi* showed higher identity with the orthologous gene of *TDF1* of *L. formosanum*, a parental species of *L*. × *formolongi*, than that of *L. longiflorum*, the other parental species, suggesting *LflTDF1* originated from *L. formosanum*. The Oriental hybrid lilies were derived from complex interspecific crosses using several species such as *L. auratum, L. nobilisimum, L. japonicum*, and *L. speciosum* (Marasek-Ciolakowska et al., 2018). Therefore, it has not yet been determined which parental species gave Siberia two *TDF1* homologs.

In this study, we successfully identified the candidate gene of the pollen sterility of *L*. × *formolongi* using RNA-seq and genetic analysis in the segregating population. Since the cross between *L*. × *formolongi* and Easter lily (*L. longiflorum*) is compatible, we successfully developed several pollen-free promising clones of Easter lily in the progenies derived from the sib cross of F_1_ plants made by crossing AR01 with Easter lily. The marker development of *TDF1* gene will assist pollen-free lily breeding of Easter lilies. In addition, we showed that the *lfltdf1* mutant never produces the pollen debris, indicating that *TDF1* orthologs are excellent target genes for producing pollen-free ornamental plants through genome editing.

## Data Availability Statement

Partial mRNA sequence of LflTDF1 was deposited to DDBJ and the accession numbers, LC704438 was assigned. Genomic sequences of Lilium TDF1 orthologs were assigned as follows: LC704885 (LflTDF1), LC704888 (LfsTDF1), LC704887 (LlTDF1a), LC704886 (LlTDF1b), LC704883 (LohTDF1a), and LC704884 (LohTDF1b).

## Author Contributions

NY, SI, and KO conceptualized the study and were involved in funding acquisition. TM, DS, NY, SI, TS, HO, HS, and SO designed methodology. TM, DS, NY, SI, TS, HO, HS, SO, and EF investigated the study. NY, SI, TS, and KO took a leading role in project administration. DS, EF, and KO reviewed and edited the manuscript. All authors contributed to the article and approved the submitted version.

## Funding

This research was supported by grants from the Project of the NARO Bio-oriented Technology Research Advancement Institution (Research program on development of innovative technology) (No. 28036C).

## Conflict of Interest

The authors declare that the research was conducted in the absence of any commercial or financial relationships that could be construed as a potential conflict of interest.

## Publisher's Note

All claims expressed in this article are solely those of the authors and do not necessarily represent those of their affiliated organizations, or those of the publisher, the editors and the reviewers. Any product that may be evaluated in this article, or claim that may be made by its manufacturer, is not guaranteed or endorsed by the publisher.
