# Supplementary material for: Identification of a Male Sterile Candidate Gene in Lilium x formolongi and Transfer of the Gene to Easter Lily (L. longiflorum) via Hybridization
Source: Front Plant Sci. 2022 Jun 29;13:914671. doi: 10.3389/fpls.2022.914671 (PMC9277459; doi:10.3389/fpls.2022.914671)
Supplement: Supplementary file 1 [file Data_Sheet_1.PDF]

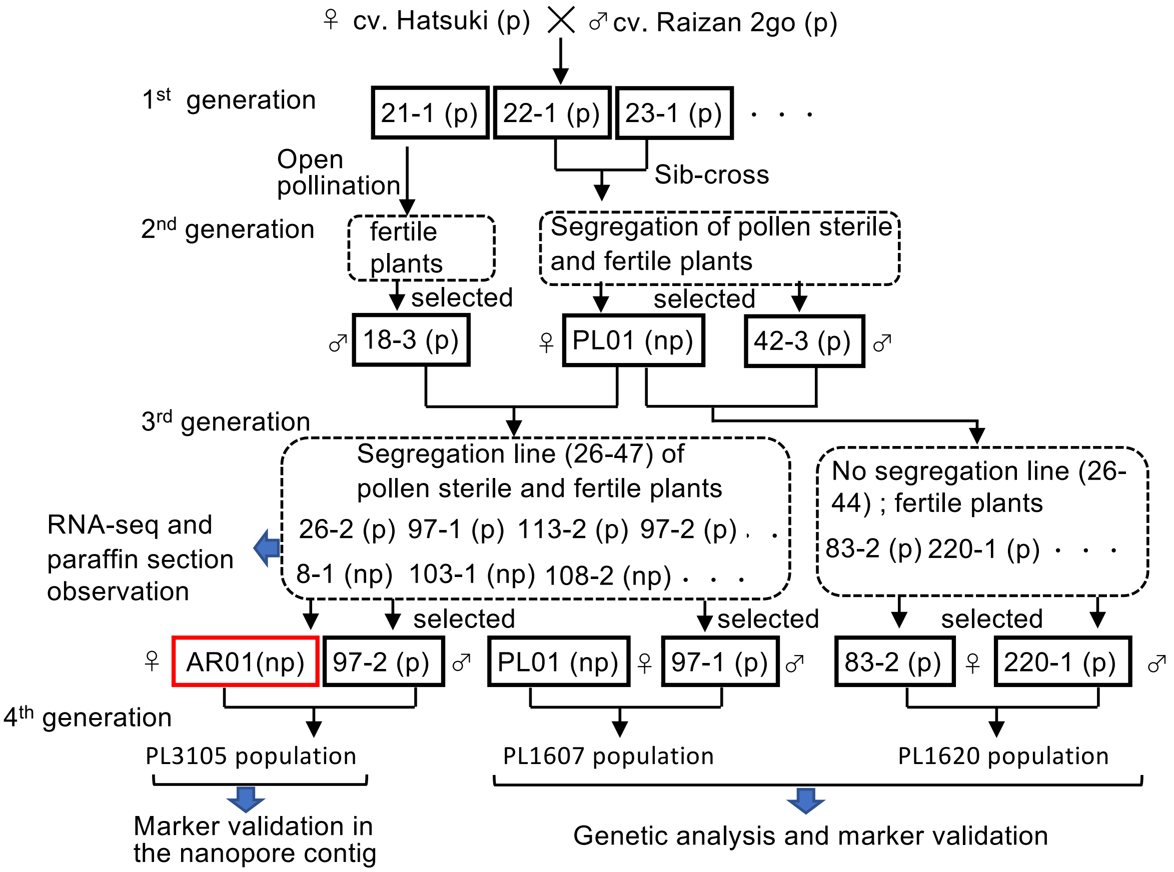

Supplementary Figure 1 Schematic diagram for development of pollen sterile cv. Akita Kiyohime (AR01) and the experimental materials. (p) and (np) represent pollen fertile and pollen sterile, respectively.
