# Supplementary material for: Identification of a Male Sterile Candidate Gene in Lilium x formolongi and Transfer of the Gene to Easter Lily (L. longiflorum) via Hybridization
Source: Front Plant Sci. 2022 Jun 29;13:914671. doi: 10.3389/fpls.2022.914671 (PMC9277459; doi:10.3389/fpls.2022.914671)
Supplement: Supplementary file 2 [file Data_Sheet_2.PDF]

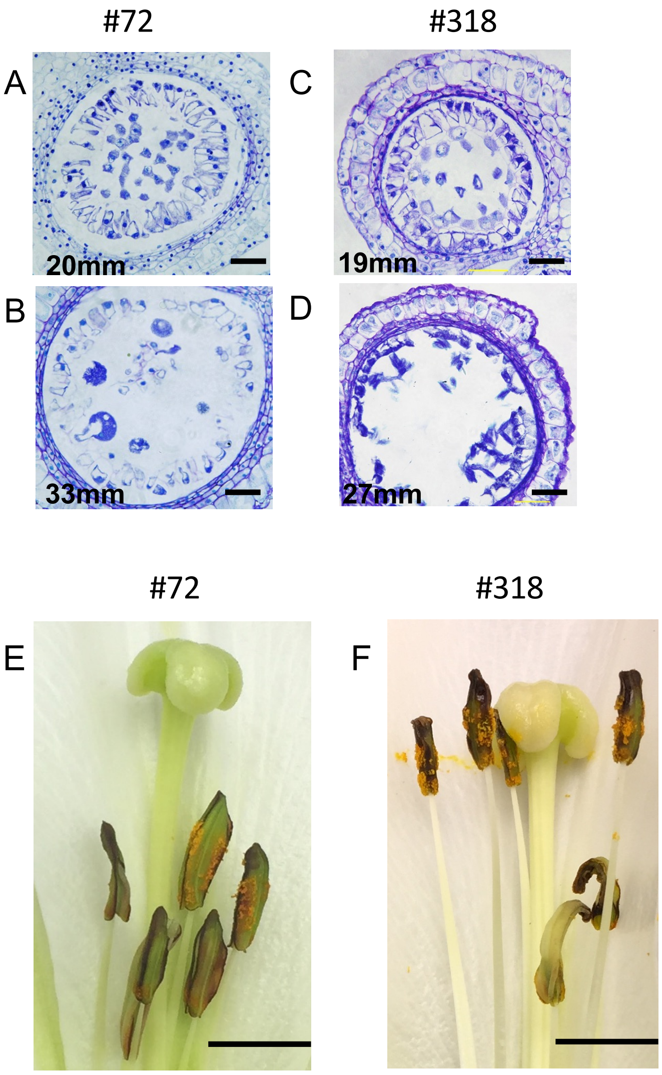

Supplementary Figure 2 Paraffin section of meiotic stage of the pollen sterile plants developed by *L.* × *formolongi* mutation breeding. (**A, B**) Pollen sterile plant, #72; (**C, D**) Pollen sterile plant, #318. Bae=100μ. Appearance of the anthers of (**E**) #72 and (**F**) #318 plants during flowering time. Bae=1 cm
