# Supplementary material for: Identification of a Male Sterile Candidate Gene in Lilium x formolongi and Transfer of the Gene to Easter Lily (L. longiflorum) via Hybridization
Source: Front Plant Sci. 2022 Jun 29;13:914671. doi: 10.3389/fpls.2022.914671 (PMC9277459; doi:10.3389/fpls.2022.914671)
Supplement: Supplementary file 3 [file Data_Sheet_3.PDF]

——：Myb –type DNA-binding domain

```
LflTDF1             MGRPPCCDKAEVRRGPWTAEEDAKLLVYTATHGTKNWTSVPKKAG-LKRCGKSCRLRWTN 59
ORUFI03G14500.1     MGRPPCCDKANVKKGPWTAEEDAKLLAYTSTHGTGNWTSVPQRAGRLKRCGKSCRLRYTN 60
AT3G28470.1-MYB35   MGRPPCCDKSNVKKGLWTEEEDAKILAYVAIHGVGNWSLIPKKAG-LNRCGKSCRLRWTN 59
                    *********::*::* ** *****:*.*.: **. **: :*::** *:*********:**

LflTDF1             YLRPNLKHDSFTLQEEELIITLHATIGSRWSIIAQQLPGRTDNDVKNIWNTKLSKKLVQR 119
ORUFI03G14500.1     YLRPNLKHENFTQEEEELIVTLHAMLGSRWSLIANQLPGRTDNDVKNYWNTKLSKKLRQR 120
AT3G28470.1-MYB35   YLRPDLKHDSFSTQEEELIIECHRAIGSRWSSIARKLPGRTDNDVKNHWNTKLKKKLMKM 119
                    ****:***:.*: :*****:  *  :***** **.:*********** *****.*** :

LflTDF1             GIDPVTHRPISEVIQSIGGLNTGASSSSSIARYRAPTDPRFSYFNQDLKSIFQSGPITAP 179
ORUFI03G14500.1     GIDPITHRPIADLMQSIGTLAIRP------------------------------PPAAG 149
AT3G28470.1-MYB35   GIDPVTHKPVSQLLAEFRNISGHG------------------------------NASFK 148
                    ****:**:*::::: .:  :                                     :

LflTDF1             GHPPWDPFPQLPSYSYCSNLSPAADEASSSSTVVTTTLSNPVAARPLSPEAEWS---DFL 236
ORUFI03G14500.1     AAPP----TCLPVFHDAPYFAALQHQHQQQQVVTHVDADAPASPDSQHLQLNWS---DFL 202
AT3G28470.1-MYB35   TEPS---NNSILTQSNSAWEMMRNTTTNHESYYTNSPMMFTNSSEYQTTPFHFYSHPNHL 205
                      *.      :     ..         . ..  .      .:.       .:    :.*

LflTDF1             VDDSFQPINENVDDAWKICSDHGKGPMVNQHELSQDNEASKILETPRSFVDEILDRDKEI 296
ORUFI03G14500.1     ADD---------------AAGHGADAPAPQAALGQYQEGSAPAAT------AVVGGGR-- 239
AT3G28470.1-MYB35   LNG----------------TTSSCSSSSSTSITQPNQVPQTPVT------NFYWSDFLL 243
                     :.                :  . ..   .  : * :: .    *       .   .

LflTDF1             LSEFPKFLNDQYGF-LVIGCKLSQNHLELSLLDFILSSFALESYETSVYLFFYFGVCEIW 355
ORUFI03G14500.1     --AFGDVDGASAG---VGAGTDDGAGAASAFIDAILD----------------------- 271
AT3G28470.1-MYB35   SDPVPQVVGSSATSDLTFTQNEHHFNIEAEYISQNID----------------------- 280
                       . .. . .     .   .          :.  :.

LflTDF1             KRNFPCDQSLSYYIFLIKFFLSLLKVNVSVTTQIMKLCFKYLRWYMKDKM 405
ORUFI03G14500.1     -----CDKEMGVDQLIAEMLADPAYYGGGGGSSSSELGWGC--------- 307
AT3G28470.1-MYB35   ------SKASGTCHSASSFVDEILDKDQEMLSQFPQLLNDFDY------- 317
```

**Supplementary Figure 3** Comparison of amino acid sequences of *TDF1* orthologs in *L.* × *formolongi*, *Oryza rufipogon* (ORUFI03G14500.1), and *Arabidopsis thaliana* (AT3G28470.1), respectively. Red line indicates Myb–type DNA-binding domain predicted by Pfam.
