# Supplementary material for: Identification of a Male Sterile Candidate Gene in Lilium x formolongi and Transfer of the Gene to Easter Lily (L. longiflorum) via Hybridization
Source: Front Plant Sci. 2022 Jun 29;13:914671. doi: 10.3389/fpls.2022.914671 (PMC9277459; doi:10.3389/fpls.2022.914671)
Supplement: Supplementary file 4 [file Data_Sheet_4.PDF]

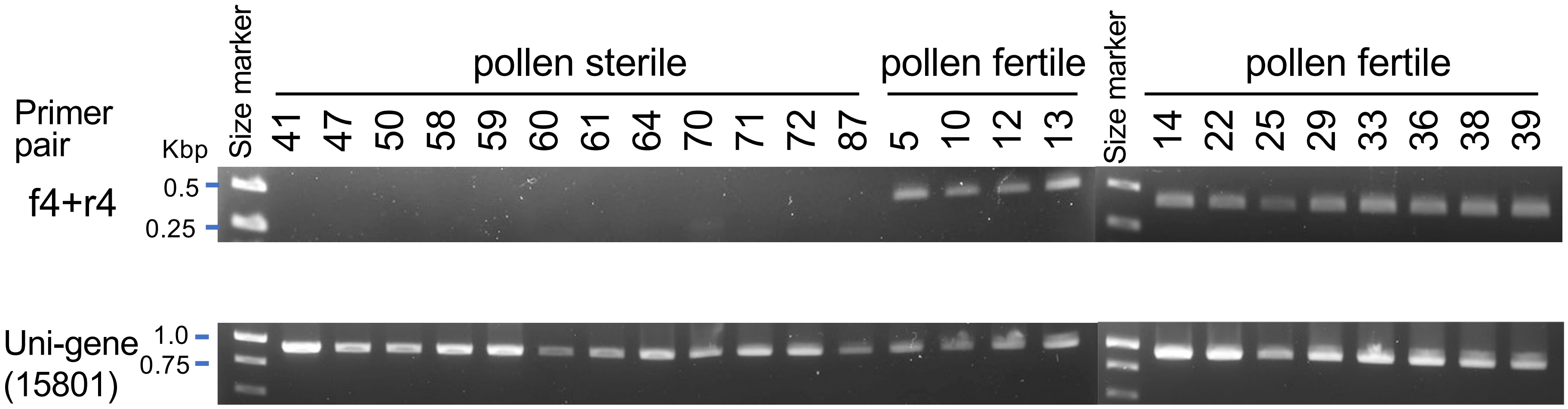

Supplementary Figure 4 An example of genotyping of LflTDF1 intragenic dominant marker, f4r4, using the segregating population (PL1607). Uni-gene (15801) primer pair was used for a positive control.
