# Supplementary material for: Identification of a Male Sterile Candidate Gene in Lilium x formolongi and Transfer of the Gene to Easter Lily (L. longiflorum) via Hybridization
Source: Front Plant Sci. 2022 Jun 29;13:914671. doi: 10.3389/fpls.2022.914671 (PMC9277459; doi:10.3389/fpls.2022.914671)
Supplement: Supplementary file 5 [file Data_Sheet_5.PDF]

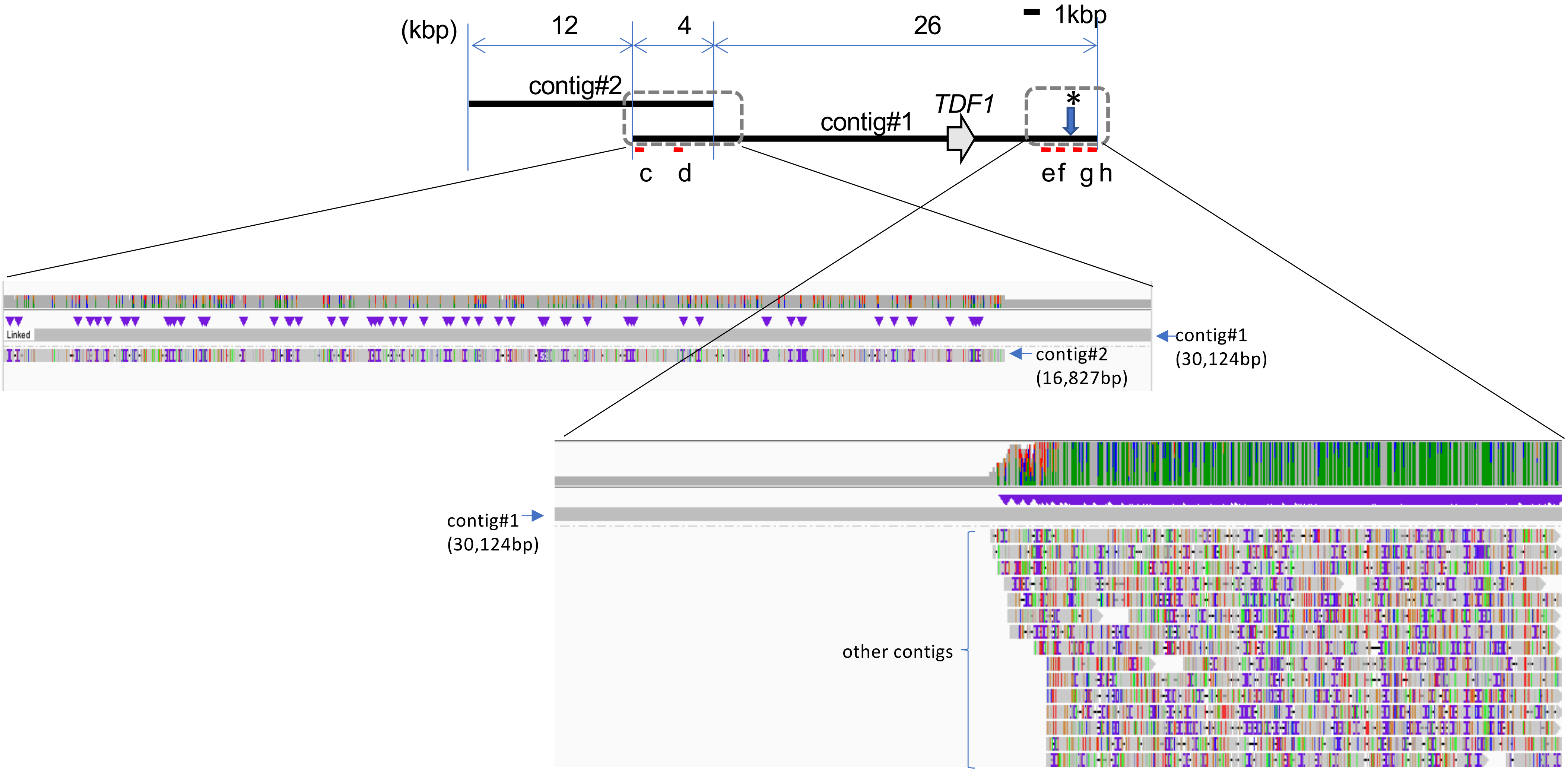

Supplementary Figure 5 Location of *LflTDF1* and nanopore sequencing contigs obtained from the fertile pollen line, N-1. Contig #1 contains *LflTDF1* gene. Contig #2 (left end) and the other contigs containing repetitive sequence (right end) overlap the homologous regions of contig #1. Striped colors and purple arrowheads indicate SNP and indels, respectively, according to IGV format. Red bars of contig #1 indicate the location of the PCR amplicons of each primer set (See Figure 5).
