# Supplementary material for: Identification of a Male Sterile Candidate Gene in Lilium x formolongi and Transfer of the Gene to Easter Lily (L. longiflorum) via Hybridization
Source: Front Plant Sci. 2022 Jun 29;13:914671. doi: 10.3389/fpls.2022.914671 (PMC9277459; doi:10.3389/fpls.2022.914671)
Supplement: Supplementary file 6 [file Data_Sheet_6.PDF]

A

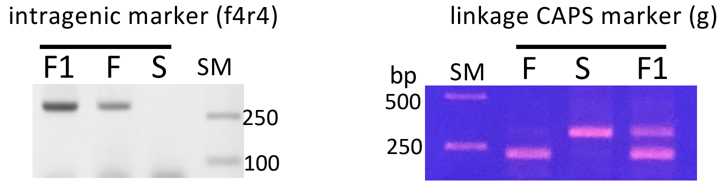

B

No, of Plants	*TDF1*-specific band with intragenic marker (f4r4)		*TDF1*-linkage CAPS marker (g)	
	Present	Absent	Cleaved	Not-cleaved
67	47	20	47	20

C

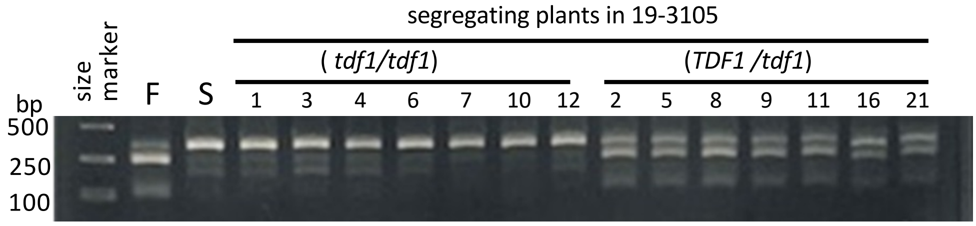

Supplementary Figure 6 PCR detection of *LflTDF1* and *lfltdf1* genotypes using the intragenic markers (f4r4) and linkage marker g. in the segregating population (PL19-3105) of AR01 (*lfltdf1*/*lfltdf1*) × 97-2 (*LflTDF1*/*lfltdf1*). (A) PCR of the fertile 97-2 (F) and sterile AR01 (S) parent and their F_1 using the intragenic markers (f4r4) and linkage marker g. (B) Segregation of *LflTDF1* and *lfltdf1* genotypes in the segregating population (PL19-3105) using the intragenic markers (f4r4) and linkage marker g. (C) An example of detection of *LflTDF1* and *lfltdf1* genotypes in the BC_1 population (PL19-3105) using the linkage marker g.
